# Managing Post-Keratoplasty Astigmatism: High-Tech vs. Low-Tech Imaging Techniques for Guiding Suture Manipulation

**DOI:** 10.3390/jcm12103462

**Published:** 2023-05-14

**Authors:** Alfredo Borgia, Vito Romano, Davide Romano, Luca Pagano, Aldo Vagge, Giuseppe Giannaccare, Mahmoud Ahmed, Kunal Gadhvi, Nardine Menassa, Mohammad Ahmad, Stephen Kaye, Giulia Coco

**Affiliations:** 1 Eye Unit, Humanitas-Gradenigo Hospital, 10153 Turin, Italy; 2 Department of Corneal Diseases, St. Paul’s Eye Unit, Royal Liverpool University Hospital, Liverpool L7 8YE, UK; 3 Eye Clinic, Department of Neurological and Vision Sciences, University of Brescia, 25125 Brescia, Italy; 4 Eye Unit, University Hospitals of Leicester, NHS Trust, Leicester LE1 5WW, UK; 5 IRCCS Ospedale Policlinico San Martino, University Eye Clinic of Genoa, 16132 Genova, Italy; 6 Department of Neurosciences, Rehabilitation, Ophthalmology, Genetics, Maternal and Child Health 19 (DiNOGMI), University of Genoa, 16132 Genova, Italy; 7 Department of Ophthalmology, University Magna Græcia of Catanzaro, 88100 Catanzaro, Italy; 8 Department of Clinical Science and Translational Medicine, University of Rome Tor Vergata, 00133 Rome, Italy

**Keywords:** corneal astigmatism, sutures adjustment, penetrating keratoplasty, deep anterior lamellar keratoplasty, autorefractometry, keratoscopy

## Abstract

Astigmatism is a visually significant condition that can develop after keratoplasty. The management of post-keratoplasty astigmatism can be performed both when transplant sutures are in place and when they have been removed. Fundamental for astigmatism management is its identification and characterization in terms of type, amount, and direction. Commonly, post-keratoplasty astigmatism is evaluated through corneal tomography or topo-aberrometry; however, many other techniques can be used in case these instruments are not readily available. Here, we describe several low-tech and high-tech techniques used for post-keratoplasty astigmatism detection in order to quickly understand if it contributes to low vision quality and to determine its characteristics. The management of post-keratoplasty astigmatism through suture manipulation is also described.

## 1. Introduction

Penetrating keratoplasty (PK) and deep anterior lamellar keratoplasty (DALK) are among the most common corneal transplant procedures performed worldwide [1]. However, despite corneal clarity, patients may still experience suboptimal vision due to postoperative high astigmatism [2,3], which occurs in up to 20% of cases [4,5].

Postoperative astigmatism is highly dependent on several aspects that can present preoperatively, intraoperatively, and postoperatively. These include the age and health of the donor tissue, the severity of the underlining disease, the shape of trephination, graft size, corneal thickness, donor–recipient disparity, a poor suturing technique, and corneal wound healing [6,7,8,9,10]. If a low postoperative astigmatism is desired, special care should be taken to a few key factors during the PK-graft; it should be round and central with an equally distributed tension of the sutures [3]. 

Two phases can be distinguished after a corneal transplant: the “plastic phase”, usually during the first 6 to 8 months after surgery, when the sutures are still in place and astigmatism can be managed by the modulation of their tension due to the transplant scar plasticity, and the “static phase” in which all sutures have been taken out, the graft scar has solidified, and the corneal architecture is no longer capable of considerable change [11]. 

The management of post-keratoplasty astigmatism can be challenging. In fact, it can be irregular and with high-order aberrations that are not completely corrigible with the use of spectacles or contact lenses [12]. Besides optical corrections, other options include postoperative suture manipulation, compression sutures, relaxing incisions [13,14,15], laser corneal surgery [16,17], intrastromal ring segment insertion [18], intraocular lens insertion (phakic or during cataract surgery) [19,20], and regrafting [21].

Today, postoperative suture manipulation is the most widely practiced technique to reduce post-keratoplasty astigmatism, either by adjusting the tension in running sutures or selectively removing interrupted sutures [22]. In fact, selective suture(s) removal on the steep axis results in a flattening of the corresponding semimeridian [23,24,25,26], thus decreasing the level of astigmatism.

Currently, the most used method to evaluate post-keratoplasty astigmatism is through corneal topography/tomography or ocular aberrometry [27]. Nevertheless, many other low-tech and high-tech techniques are available to aid the evaluation of post-keratoplasty astigmatism.

This review aims to describe the available low-tech and high-tech solutions to guide ophthalmologists in the diagnosis and management of post-keratoplasty astigmatism by suture manipulation.

## 2. The Role of Low-Tech in Post-Keratoplasty Astigmatism Detection

In this section, the role of low-tech in post-keratoplasty astigmatism detection, including slit lamp biomicroscopy, keratoscopy, keratometry, retinoscopy, and manifest refraction is explored. 

### 2.1. Slit Lamp Biomicroscopy

**Figure 1:**
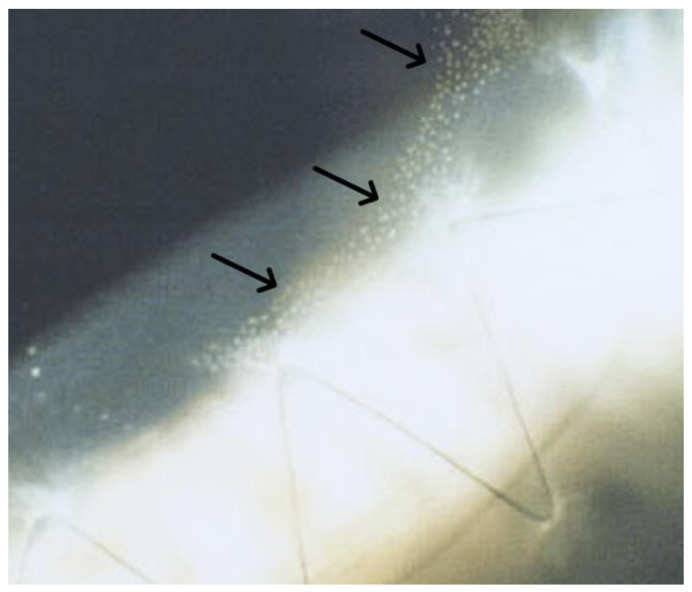
White punctate epithelial opacities (black arrows) known as “Kaye dots” present anterior to the suture line of a corneal graft (PK) in the epithelium.

Slit lamp examination is still essential and represents the gold standard for the postoperative evaluation of a corneal transplant [28]. Slit lamp is used to assess graft size and centration [29]; larger grafts are associated with less astigmatic changes compared with smaller ones. Additionally, a decentered graft may cause irregular astigmatism [30,31]. The graft–host interface should be inspected for apposition quality (override or underride) and surgical wound stability. Sutures should be examined to detect loose or excessively tight sutures. If any corneal area is grossly steeper than others, this can be visible through lines on the front corneal surface. Slit lamp biomicroscopy findings associated with tight sutures are usually related to keratoscopic characteristics. An elevated doughnut of compressed tissue between sutures indicates a wound area that has been intensively compressed [9]. The presence of Kaye’s epithelial dots (Figure 1), which first appear central to the suture line, may be associated with the elevated doughnut [32]. Kaye’s dots are related to tight sutures and may represent abnormal epithelial healing and be associated with an iron line in the affected quadrant [32]. These dots, usually, rapidly disappear once sutures are removed, except in the case of subepithelial fibrosis developed prior to suture removal in the same area. In fact, over time, tight sutures may determine subepithelial fibrosis and, if severe, keratoscopic examination may show a flattening in this area [33]. An animal study on rabbits showed that a horizontal mattress suture (HMS) determined more fibrosis than a simple interrupted suture (SIS) in the early stages of corneal wound healing, even though equivalent results were obtained after three months [34]. 

Tight sutures may also result in stress lines at the level of the Descemet’s membrane or Bowman’s layer spreading in its direction [9].

### 2.2. Keratoscopy

**Figure 2:**
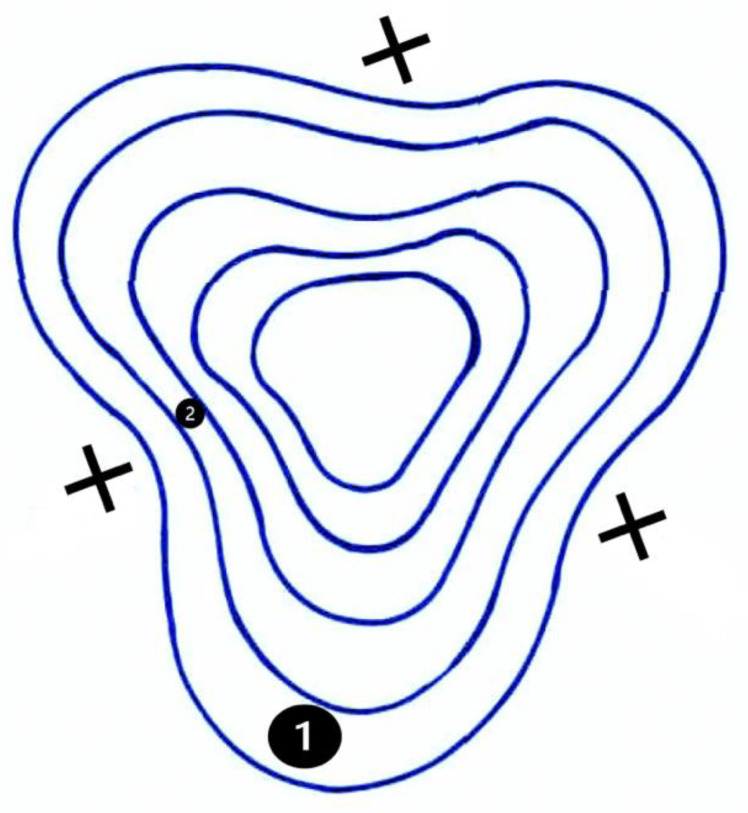
Schematic illustration of a keratoscopy with three tight sutures (black cross symbols) following keratoplasty. This results in the formation of a Trefoil HOA; the three main compression vectors are placed in a triangular configuration. Widely spaced rings suggest low corneal power (1); closely spaced rings are an indication of a higher corneal power (2).

A small hole in the centre of the disk allows the examiner to see the reflection of a set of concentric rings that are projected onto the cornea. Although most keratoscopies performed today are the ones integrated with high-tech devices such as advanced autorefractometers, corneal topographers, and corneal tomographers (Scheimplug or OCT-based), a simple keratoscopy can still have a role in the ocular surface and corneal aberrations analysis, especially in circumstances where other high-tech tools are not available. Radial distance is determined by the position of the ring in relation to the pattern’s centre. The curvature radii depend on the size of the rings and their distance from each other. High corneal power is indicated by closely spaced rings, while low corneal power is indicated by widely spaced rings (Figure 2) [35]. Photokeratoscopy indicates the process of photographing keratoscopy pictures. During photokeratoscopy, a keratoscope is used to display lit images onto the corneal surface. It makes the cornea’s concentric rings clearly visible. This offers a qualitative assessment of the surface. A steeper cornea, rather than a flatter one, will reflect mires that are thinner and more tightly spaced; additionally, mires in the steep area are nearer to the center. An irregular surface reflects distorted and irregular mires [36]. 

During videokeratoscopy, the evaluation is recorded with a camera. The aforementioned technologies do not offer quantitative information on corneal surface or power. Interpretation is impacted by mucus, tear film alterations, and particles in the tears [37]. To minimize artifacts, central mires must be positioned parallel to the corneal apex.

### 2.3. Keratometry

Manual keratometers calculate the corneal radius of curvature at the central 3-mm zone, estimating the curvature radius and the dioptric power with the aid of mire size measurements. Two keratometry readings are sufficient to determine the central corneal curvature and power on a regular corneal surface. However, its application is rudimentary for irregular ocular surfaces. Due to surface irregularity and wound healing, assessing astigmatism through keratometry in the early postoperative period may be challenging [38]. Furthermore, since keratometry presumes that the corneal surface is spherocylindrical, it cannot measure the ocular surface properly if this is not spherocylindrical. Additionally, only the radius of curvature between two points that are 3 mm apart is measured. Hirst et al. demonstrated that central keratometry may effectively allow for post-keratoplasty astigmatism evaluation and decision-making about suture manipulation [39]. However, keratometry offers no information on locations inside or outside of these two places and the paracentral and peripheral surfaces are not examined in detail. Furthermore, irregular astigmatism and high order aberrations (HOA) are not quantified. Modern automated keratometers are integrated in high-tech devices such as optical biometers and sophisticated autorefractometers.

### 2.4. Retinoscopy

**Figure 3:**
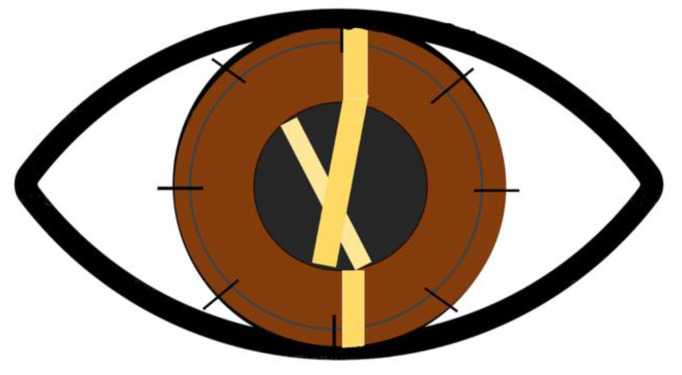
Schematic illustration of a “Scissor Effect” during retinoscopy in a post keratoplasty eye with tight sutures. In case of irregular astigmatism, it is possible to appreciate the two bands moving toward and away from one another during retinoscopy like the blades of scissors.

The basic principle of retinoscopy is to get the patient’s distant point close to the observer’s nodal point. Refractive power and the main meridians are both identified using a streak retinoscope or a plane mirror. Complex astigmatism that is present after corneal transplants is usually difficult to identify with this technique, due to corneal irregularity and the presence of HOA. These can produce a scissoring effect in the area of steepening (Figure 3), similar to the one observed in the advanced keratoconus, and the retinoscopy reflex will spin in a manner that is characteristic for corneal irregularity. Tight sutures cause residual astigmatism, and the retinoscopy beam’s narrowest side is focused directly on the tightest suture [9].

### 2.5. Manifest Refraction

Post-keratoplasty astigmatism detection allows for the identification of the best corrected vision in the eye. Patient dissatisfaction following surgery is frequently caused by the subjective loss of visual quality, which can typically be diagnosed by understanding how corneal shape abnormalities affect the optics of the eye. Ideally, in post-keratoplasty patients, the manifest refraction should be measured in photopic, mesopic, and scotopic conditions, in consideration of the highly aberrated corneas which can determine symptoms depending on the pupil size [40,41].

An automated refraction with an autorefractometer is useful in determining the manifest refraction starting point. Subjective refraction refined with a phoropter, or trial lens set, is recommended in cooperative patients. Distance refraction should be examined with accommodation relaxed, employing manifest (noncycloplegic) refraction with fogging or other techniques to minimize accommodation, and taking care not to give the patient excessively negative minus power correction. Cycloplegic refraction may be beneficial, especially in young phakic patients, to better evaluate the spherical equivalent.

Patients who wear therapeutic or rigid contact lenses should stop wearing them before the evaluation, since contact lenses can cause corneal warpage and inconsistent refractive measurements. As a general rule, spherical soft contact lenses should be removed for at least 3 days to 2 weeks prior, whereas rigid/semiscleral/scleral should be removed for up to 6 weeks before evaluation [42].

Refraction alone often detects the flat or the steep meridian only. Nevertheless, due to the complex astigmatism after keratoplasty, it is usually necessary to perform further testing in addition to refraction to precisely identify the steep and flat areas [43]. In addition, differing from keratometry and corneal topography, refraction is influenced by the posterior cornea [44] and internal aberrations (lenticular astigmatism, tilted lens, posterior segment alterations) [45]; this aspect should be considered before suture manipulation, especially in cases where these factors are modifiable over time.

## 3. The Role of High-Tech in Post-Keratoplasty Astigmatism Detection

In this section, the role of high-tech in post-keratoplasty astigmatism detection, including autorefractometry with keratoscopy, Placido disk-based topography (topography), rotating Scheimpflug photography (tomography), anterior segment optical coherence tomography (AS-OCT), and topo-aberrometry, is discussed.

### 3.1. Auto-Refractometry Associated with Keratoscopy

The autorefractometer associated with a keratoscope, which is often available in the outpatient clinic, may be considered a viable option, especially in cases of fairly regular post-keratoplasty astigmatism. Analysing the auto-refractometer results in combination with the integrated keratoscopy, it is possible to deduce the anterior corneal morphology [46]. As mentioned in the keratoscopy section, the closer the keratoscopic rings are, the higher the curvature of the analysed surface; on the other hand, rings far apart indicate a flat surface [35]. One of the advantages of the autorefractometer is its ability to detect the overall astigmatism of the patient’s eye. This can be useful in cases where lenticular astigmatism or IOL tilting have an impact on the overall eye refraction; thus, to improve patients’ vision, corneal curvature might not be the only factor to consider. In post-keratoplasty astigmatism, it has been shown that when the axis of astigmatism identified with topography and autorefractometry coincide, astigmatism management through suture removal has better results compared with incidences when the results of these exams are in disagreement [45]. 

In cases of irregular astigmatism or in the presence of high order aberrations, the rings may be irregular or even absent in some areas where sudden changes are present. Topography in such cases represents a better option to identify the steep axis [47].

### 3.2. Placido Disk-Based Topography

Color maps depicting anterior corneal power and elevation are produced using this tool. In comparison with refraction, corneal topography has the benefit of precisely detecting small dioptric variations throughout the anterior cornea’s optical zone and revealing surface irregularities. Additionally, using topography, it is possible to identify steep peripheral areas commonly present after corneal transplant [47]. According to Maguire and Bourne, the idea of the steep hemimeridians seen on corneal topography is a better way to understand the steep axis in post-PKP grafts [47]. The hemimeridians commonly have power asymmetry and are spaced from each other at an angle different from 180 degrees. Areas of maximal steepening are clearly visible with topography, as well as those that are peripheral or in locations other than the major steep hemimeridians [47]. In the case of irregular astigmatism, topography has shown to be superior to refraction and keratometry [45,48]. The effect of suture removal is greatest when the steep axis of the astigmatism measured by topography and the steep axis determined by refraction coincide [45]. However, the astigmatism determined by refraction and topography may vary significantly in cases of lenticular astigmatism [49]. According to Sarhan et al., after suture manipulation, there is a significant decrease in manifest refraction, keratometry, and topographical astigmatism if the difference in the axes of astigmatism between those is within 11 degrees [45]. Additionally, Sarhan et al. showed that corneal topography carried out 30 to 45 min after suture removal accurately predicted the subsequent set of sutures to be removed for the residual astigmatism [23]. Consequently, patients who had corneal grafts might have a second set of sutures removed at the same follow-up appointment [23]. However, corneal topography only assesses the front surface of the cornea.

### 3.3. Scheimpflug Photography (Tomography)

In Scheimpflug imaging, corneal cross-sections produced by slit beams at various angles are captured on a camera film. This technology adjusts for the cornea’s non-planar form, enabling the creation of a three-dimensional map of the cornea with higher precision and resolution. In a single scan, the rotational Scheimpflug camera captures up to 50 cross-sectional images at angles ranging from 0 to 180°, collecting 25,000 data points in a few seconds [50]. The second camera is stationary and is used to measure pupil size and track eye fixation. The integrated technology employs an algorithm to adjust for any picture distortion produced. The rotating camera allows for the acquisition of accurate measurements of irregular corneas, which reflect when Placido-based sensors find difficulties in capturing. Corneal tomography obtains information regarding corneal curvature across the entire cornea by measuring the shape of the anterior and posterior surfaces. The integrated software displays axial, instantaneous, and elevation maps of the anterior and posterior surface topography, pachymetry data from limbus to limbus, and analyses of the anterior chamber and lens. 

During the analysis of the post-transplant corneal morphology and the donor-recipient junction, it is essential to analyse the anterior elevation areas that can cause low and high order aberrations and understand their origin before performing suture manipulation. In particular, it is necessary to differentiate conditions in which there is a bulging at the junction determined by an initial localised diastasis of the graft from conditions in which there is instead an increased anterior elevation due to tissue deposition (corneal epithelium) or following localised endothelial dysfunction. To determine the origin of this alteration in tomographic maps, it is fundamental to evaluate corneal pachymetry and posterior elevation in the same area. In cases of initial diastasis, there is typically correspondence between localised pachymetric thinning, increased posterior elevation, and a possible increase in the anterior elevation at the same location. While in cases of focal endothelial dysfunction or in the case of increased tissue apposition (corneal epithelium) at the donor–recipient junction, due to the high corneal curvature gradient [36], there will be a pachymetric increase without the associated posterior elevation at the same point. The use of epithelial maps and the analysis of the corneal surface via slit lamp often help in differentiating the last two conditions [51].

**Figure 4:**
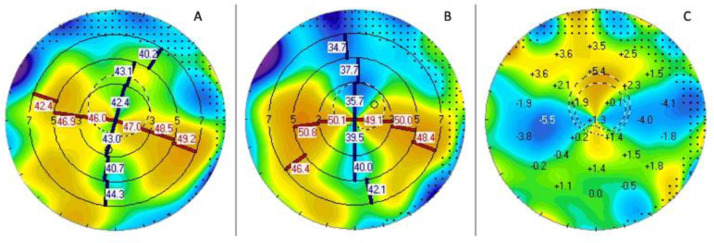
Scheimbflug tomography (Pentacam, Oculus Systems, Wetzlar, Germany) analysis of axial map pre (**B**) and post (**A**) selective suture removal, and differential map (**C**). The patient’s initial astigmatism, 7 months after a PK for corneal scarring, was 11.7 D of astigmatism at 176°. The sutures at 3 and 9 o’clock were selectively removed and the astigmatism was reduced to 3.9 D at 156° after 3 months. The autorefractometry was −1.75 +3 @155 and patient achieved a visual acuity of 0.1 LogMar.

Chaurasiya et al. investigated the amount and direction of post-keratoplasty astigmatism changes following suture removal (SR), comparing refraction, tomography, and aberrometry [27]. Their study showed that the pattern of astigmatism change, as determined by various instruments, might vary. Additionally, corneal tomography resulted in being the primary diagnostic tool used to track astigmatism evolution following SR [27]. Fares et al. demonstrated a decrease in postoperative astigmatism up to 2.6 D after Scheimpflug topography-guided suture manipulation [28]. Accordingly, Chaurasiya et al. have found a similar trend with a mean decrease of 2.43 D and 2.17 D of tomographic and refractive astigmatism, respectively [27]. One example is shown in Figure 4 with a change of astigmatism from 11.7 to 3.9 after a selective removal of two sutures.

There are certain benefits of using near-infrared light over a visible light source. Biological tissues are known to greatly scatter light in the visible spectrum. Additionally, due to its intense absorption, visible light cannot pass through human tissue. In contrast, since near-infrared waves have minimal tissue absorption and scattering properties, they are far more permeable across biological media than shorter visible light waves [52].

### 3.4. Anterior Segment Optical Coherence Tomography (AS-OCT)

Anterior segment OCT has a relatively long wavelength that enables it to penetrate relatively deep even in non-transparent tissues (corneal infiltrates, scarring, and opacity) [53,54,55], and it can attain micrometer resolution [56,57].

Graft–host junction misalignment, irregular or delayed wound healing, or unequal suture tension can all result in regular or irregular astigmatism that causes visual distortion. Slit lamp examination is likely to miss internal junction misapposition because, typically, it is not detected on the front surface [58,59]. With special attention to the internal alignment of the graft–host junction, AS–OCT has been utilized to analyse the configuration of the graft–host junction and assess wound healing [58,59,60,61]. Primary diagnosis and graft–host oversizing can affect the misalignment of the graft-host junction [58,59,60]. When the thickness between the donor and recipient is different, there will be a step-like graft–host junction with an excess of donor or host corneal tissue extending towards the anterior chamber [58,59,60]. In case of endothelial dysfunction, a sudden posterior corneal curvature change could be evident; this may happen when the edema in the host cornea persists after the transplant [58,59]. According to Szalai et al., when compared with the high-resolution Scheimpflug system, swept-source OCT (SS-OCT) offers more accurate anterior segment evaluations in patients who had penetrating keratoplasty [62].

#### Corneal Epithelial Mapping

Modern corneal epithelium thickness measurement can be obtained using corneal tomographic devices such as RTVue (Optovue Inc, Fremont, CA, USA), MS-39 OCT (CSO, Florence, Italy), Cirrus HD OCT (Carl Zeiss Meditec, Jena, Germany), CASIA-2 (Tomey, Aichi, Japan), or using the Artemis very high-frequency (VHF) digital ultrasound system (ArcScan Inc, Morrison, CO, USA) [51,63,64]. 

It was discovered that variations in the epithelial thickness profile are very predictable, reacting to alterations in the gradient of the stromal curvature. Since the epithelia partially hide stromal surface irregularities [36,65], adaptive epithelial thickness alterations are thus a crucial component of the diagnosis of irregular astigmatism [66]. The epithelial thickness map can represent an additional tool in the evaluation of irregular astigmatism post-keratoplasty. In particular, with these maps it is possible to analyse corneal epithelial distribution due to really steep or flat areas, or ocular surface abnormalities, which can affect post-keratoplasty refraction.

### 3.5. Topo-Aberrometry

Topo-aberrometry is a highly reliable and operator-independent tool for obtaining an exact objective refraction, which is crucial in the management of post-keratoplasty astigmatism. Wavefront mapping helps us understand the patient’s visual quality and individuates possible internal aberrations compensating corneal irregularities and has been proved a valid tool to evaluate post-keratoplasty astigmatism [27]. 

Furthermore, topo-aberrometry is particularly important in post-keratoplasty eyes with anomalies in the iris, lens, and IOL-bag complex. In addition, this tool plays a key role in the correction of astigmatism and in the development of new technological contact lenses capable of significantly reducing the perception of HOA [67].

## 4. The Role of AI High-Tech in the Post-Keratoplasty Astigmatism Detection

Artificial intelligence (AI) has been used to diagnose and forecast several corneal conditions, including problems from corneal grafts, keratoconus, and irregular astigmatism [68]. AI technology can analyse corneal topography data and provide highly accurate and objective measurements of astigmatism after keratoplasty. Additionally, AI algorithms can analyse large datasets and extract patterns, allowing for the precise identification of astigmatic changes in the cornea. Furthermore, AI algorithms can evaluate corneal topography data over time and identify changes in astigmatism. This monitoring can enable early intervention and the modification of treatment plans if necessary. Modern software included in corneal tomography and topo-aberrometry permit the analysis of the ocular wavefront and understand the quality of vision after keratoplasty, improving the ability to correct HOAs in addition to sphere and cylinder (LOAs) [69].

## 5. Management of Post-Keratoplasty Astigmatism: Sutures Manipulation

After the evaluation of corneal astigmatism, postoperative suture manipulation represents the most common method of reducing postoperative astigmatism [28]. The rationale of suture manipulation is based on the principle that astigmatism may be induced by excessively tight sutures, so that removing one or more tight sutures can lead to a reduction in the overall astigmatism [25,70].

However, before performing a suture removal, it is important to evaluate the effect of all sutures, as in some cases, a very tight suture may have a corresponding tight suture 180° away. Therefore, the removal of one suture may amplify the effect of the other tight suture on the astigmatism. 

An evaluation of refraction and corneal morphology is suggested to guide suture removal. Refraction and keratometry, manual or automated, identify only the steep and flat corneal meridians 90° apart, whereas the corneal topography/tomography can identify one or more steep hemi-meridians which are not necessarily 180° apart. Corneal topography/tomography also has the advantage of accurately mapping fine corneal power changes over the entire optical zone and beyond, allowing for the identification of steep meridians that can be attributed to specific sutures. However, it does not consider internal astigmatism and aberrations, which are detected through a topo-aberrometric analysis, which ultimately provides the most accurate method. Nevertheless, the latter requires specific devices, which are usually expensive and not readily available in most outpatient clinics. After having identified the steep axis, if interrupted sutures are in place, the sutures on the steepest axis (or a single suture on the steepest hemi meridian) are removed, leading to a flattening in the corresponding meridian/hemi-meridian [43]. The patient is then re-evaluated a few weeks after, with the possible removal of other sutures [44]. While in the case of continuous sutures, tension through a continuous suture is modified by loosening the suture in the steep meridian and tightening it in the flat meridian, leading to a flattening of the steep meridian [4,43]. 

This approach is cheap, not time-consuming, and can be performed at the slit-lamp. It can be performed either early in the postoperative period (between 1 and 3 months) in cases of combined interrupted and continuous sutures or double-continuous sutures, or later (between 3 and 6 months) in the case of interrupted sutures [44]. 

There is no clear indication on how long after the corneal transplant the astigmatism should be corrected. However, in most patients, six-month post-keratoplasty is considered an acceptable time for selective suture removal (SSR) [71,72]. Indeed, given the avascularity of the corneal stroma, early suture removal may lead to graft wound dehiscence [72], while delaying it for too long may decrease its impact on the astigmatism [71].

When single suture removal is not enough for the astigmatism correction, nonadjacent sutures can be removed at intervals of 4–6 weeks [24,26]. Comparing SSR versus multiple sutures removal at a time, the first one is a better option as multiple sutures removal may cause refractive surprise with a corresponding worsening of vision [43,73,74]. 

If combined running and interrupted sutures are present, SSR can be performed in the 5 months post-keratoplasty [74].

Conversely, in the case of continuous sutures, their adjustment, loosening the suture in the steep meridian and tightening it in the flat meridian, reduces the astigmatism more effectively if performed intraoperatively or in the first 2 weeks after the transplant [73].

## 6. Conclusions

Suture manipulation is the most common method for reducing post-keratoplasty astigmatism during the plastic phase. For the identification of post-keratoplasty astigmatism, the use of several techniques and devices is crucial. However, refraction, keratometry, and topography/tomography are sometimes in disagreement. A consensus among the various approaches indicates a better prognosis after suture removal, with a larger vector-corrected change, and a larger net astigmatism reduction. The best devices available in terms of accuracy and reliability should be used for the preoperative evaluation of suture manipulation to achieve the best outcome. Slit lamp evaluation and subjective refraction remain essential in the post-keratoplasty assessment, even with the advent of new high-tech devices. Anterior corneal curvature is best described by Placido disk topography and tomography. Corneal shape is more precisely described by different tomographers (Scheimplug and OCT based). Finally, more recent methods are employed to assess corneal irregularities, including Fourier analysis and Zernike’s polynomials, and AS–OCT with epithelial corneal mapping. In the case of regular astigmatism, the autorefractor associated with keratoscopy can be a viable solution in guiding suture removal/adjustment, when more advanced instruments are not available, while in the case of irregular astigmatism, a corneal tomographer or topo-aberrometer remain the preferred choices.

## Data Availability

Not applicable.
